# Supplementary material for: Influence of Fixation Methods on Prosthetic Joint Infection Following Primary Total Knee Replacement: Meta-Analysis of Observational Cohort and Randomised Intervention Studies
Source: J Clin Med. 2019 Jun 11;8(6):828. doi: 10.3390/jcm8060828 (PMC6616920; doi:10.3390/jcm8060828)

<u>Supplementary Material</u>

**Supplementary Table S1**	PRISMA checklist
**Supplementary Table S2**	MOOSE checklist
**Supplementary Table S3**	Literature search strategy
**Supplementary Table S4**	Reference list of included studies
**Supplementary Figure S1**	Assessment of risk of bias in randomised controlled trials
**Supplementary Figure S2**	Comparison of all uncemented fixation with cemented fixation and the risk of prosthetic joint infection in observational studies
**Supplementary Figure S3**	Comparison of hybrid fixation with uncemented or all cemented fixations and the risk of prosthetic joint infection in observational studies
**Supplementary Figure S4**	Comparison of antibiotic-loaded cemented fixations with plain cemented fixations and the risk of prosthetic joint infection in observational studies
**Supplementary Figure S5**	Comparison of uncemented fixation with cemented or hybrid fixations and the risk of prosthetic joint infection in randomised controlled trials
**Supplementary Figure S6**	Assessment of small study effects by funnel plots and Egger's regression symmetry tests

Supplementary Table S1. PRISMA checklist

Section/topic	Item No	Checklist item	Reported on page No
**Title**			
Title	1	Identify the report as a systematic review, meta-analysis, or both	1
**Abstract**			
Structured summary	2	Provide a structured summary including, as applicable, background, objectives, data sources, study eligibility criteria, participants, interventions, study appraisal and synthesis methods, results, limitations, conclusions and implications of key findings, systematic review registration number	2
**Introduction**			
Rationale	3	Describe the rationale for the review in the context of what is already known	4-5
Objectives	4	Provide an explicit statement of questions being addressed with reference to participants, interventions, comparisons, outcomes, and study design (PICOS)	5
**Methods**			
Protocol and registration	5	Indicate if a review protocol exists, if and where it can be accessed (such as web address), and, if available, provide registration information including registration number	2
Eligibility criteria	6	Specify study characteristics (such as PICOS, length of follow-up) and report characteristics (such as years considered, language, publication status) used as criteria for eligibility, giving rationale	6
Information sources	7	Describe all information sources (such as databases with dates of coverage, contact with study authors to identify additional studies) in the search and date last searched	6
Search	8	Present full electronic search strategy for at least one database, including any limits used, such that it could be repeated	Supplementary Table S3
Study selection	9	State the process for selecting studies (that is, screening, eligibility, included in systematic review, and, if applicable, included in the meta-analysis)	6-7
Data collection process	10	Describe method of data extraction from reports (such as piloted forms, independently, in duplicate) and any processes for obtaining and confirming data from investigators	6-7
Data items	11	List and define all variables for which data were sought (such as PICOS, funding sources) and any assumptions and simplifications made	6-7
Risk of bias in individual studies	12	Describe methods used for assessing risk of bias of individual studies (including specification of whether this was done at the study or outcome level), and how this information is to be used in any data synthesis	7-8
Summary measures	13	State the principal summary measures (such as risk ratio, difference in means).	7-8
Synthesis of results	14	Describe the methods of handling data and combining results of studies, if done, including measures of consistency (such as I^2 statistic) for each meta-analysis	7-8
Risk of bias across studies	15	Specify any assessment of risk of bias that may affect the cumulative evidence (such as publication bias, selective reporting within studies)	7-8
Additional analyses	16	Describe methods of additional analyses (such as sensitivity or subgroup analyses, meta-regression), if done, indicating which were pre-specified	7-8
**Results**			
Study selection	17	Give numbers of studies screened, assessed for eligibility, and included in the review, with reasons for exclusions at each stage, ideally with a flow diagram	8 and Figure 1
Study characteristics	18	For each study, present characteristics for which data were extracted (such as study size, PICOS, follow-up period) and provide the citations	8-9, Table 1
Risk of bias within studies	19	Present data on risk of bias of each study and, if available, any outcome-level assessment (see item 12).	9-10, Table 1; Supplementary Figure S1
Results of individual studies	20	For all outcomes considered (benefits or harms), present for each study (a) simple summary data for each intervention group and (b) effect estimates and confidence intervals, ideally with a forest plot	9-10, Figures 2-3
Synthesis of results	21	Present results of each meta-analysis done, including confidence intervals and measures of consistency	9-10, Figure 2; Supplementary Figures S2-S5
Risk of bias across studies	22	Present results of any assessment of risk of bias across studies (see item 15)	Figure 3
Additional analysis	23	Give results of additional analyses, if done (such as sensitivity or subgroup analyses, meta-regression) (see item 16)	9-10; Figure 3
**Discussion**			
Summary of evidence	24	Summarise the main findings including the strength of evidence for each main outcome; consider their relevance to key groups (such as health care providers, users, and policy makers)	10
Limitations	25	Discuss limitations at study and outcome level (such as risk of bias), and at review level (such as incomplete retrieval of identified research, reporting bias)	13-14
Conclusions	26	Provide a general interpretation of the results in the context of other evidence, and implications for future research	12-14
**Funding**			
Funding	27	Describe sources of funding for the systematic review and other support (such as supply of data) and role of funders for the systematic review	14

Supplementary Table S2. MOOSE checklist

Influence of fixation methods on prosthetic joint infection following primary total knee replacement: meta-analysis of observational cohort and randomised intervention studies

Criteria		Brief description of how the criteria were handled in the review
**Reporting of background**		
√	Problem definition	Prosthetic joint infections (PJIs) though uncommon, are dreaded and devastating complications of total joint replacements. Whether implant-related factors such as the fixation method influences the risk of infection following total knee replacement (TKR) is a contentious issue. In this context, we have carried out a systematic review and meta-analysis to evaluate the body of evidence linking fixation methods (cemented, uncemented, and hybrid) with the risk of PJI following TKR.
√	Hypothesis statement	Fixation techniques which include cemented, uncemented, and hybrid may be associated with the risk of periprosthetic joint infection (PJI) following TKR.
√	Description of study outcomes	Periprosthetic joint infection
√	Type of exposure	Cemented, uncemented, and hybrid, and fixations
√	Type of study designs used	Comparative observational studies and randomised controlled trials
√	Study population	Patients followed for PJI outcomes following TKR
**Reporting of search strategy should include**		
√	Qualifications of searchers	Setor K. Kunutsor, PhD; Vikki Wylde, PhD
√	Search strategy, including time period included in the synthesis and keywords	Time period: from inception to November 2018<br>The detailed search strategy can be found in Supplementary Table S3
√	Databases and registries searched	MEDLINE, EMBASE, Web of Science, and Cochrane databases
√	Search software used, name and version, including special features	OvidSP was used to search EMBASE and MEDLINE<br>EndNote 11 used to manage references
√	Use of hand searching	We searched bibliographies of retrieved papers
√	List of citations located and those excluded, including justifications	Details of the literature search process are outlined in the flow chart. The citation list for excluded studies are available on request.
√	Method of addressing articles published in languages other than English	Not applicable
√	Method of handling abstracts and unpublished studies	Abstracts with no full text publications were not included.
√	Description of any contact with authors	None
**Reporting of methods should include**		
√	Description of relevance or appropriateness of studies assembled for assessing the hypothesis to be tested	Detailed inclusion and exclusion criteria are described in the Methods section.
√	Rationale for the selection and coding of data	Data extracted from each of the studies were relevant to the population characteristics, study design, exposure, and outcome.
√	Assessment of confounding	We assessed confounding by ranking individual studies on the basis of different adjustment levels and performed sub-group analyses to evaluate differences in the overall estimates according to levels of adjustment.
√	Assessment of study quality, including blinding of quality assessors; stratification or regression on possible predictors of study results	Study quality was assessed based on the nine-star Newcastle–Ottawa Scale using pre-defined criteria namely: population representativeness, comparability (adjustment of confounders), ascertainment of outcome. Sensitivity analyses by several quality indicators such as study size, duration of follow-up, and adjustment factors.
√	Assessment of heterogeneity	Heterogeneity of the studies was quantified with I^2 statistic that provides the relative amount of variance of the summary effect due to the between-study heterogeneity and explored using meta-regression and stratified analyses
√	Description of statistical methods in sufficient detail to be replicated	Description of methods of meta-analyses, sensitivity analyses, meta-regression and assessment of publication bias are detailed in the methods. We performed random effects meta-analysis with Stata 15.
√	Provision of appropriate tables and graphics	Table 1; Figures 1-3; Supplementary Figures S1-S6
**Reporting of results should include**		
√	Graph summarizing individual study estimates and overall estimate	Supplementary Figures S1-S5

√	Table giving descriptive information for each study included	Table 1
√	Results of sensitivity testing	Sensitivity analysis was conducted to assess the influence of some large studies and low-quality studies on the pooled estimate.
√	Indication of statistical uncertainty of findings	95% confidence intervals were presented with all summary estimates, I^2 values and results of sensitivity analyses
**Reporting of discussion should include**		
√	Quantitative assessment of bias	Sensitivity analyses indicate heterogeneity in strengths of the association due to most common biases in observational studies. The systematic review is limited in scope, as it involves published data. Individual participant data is needed. Limitations have been discussed.
√	Justification for exclusion	All studies were excluded based on the pre-defined inclusion criteria in methods section.
√	Assessment of quality of included studies	Brief discussion included in 'Methods' section
**Reporting of conclusions should include**		
√	Consideration of alternative explanations for observed results	Discussion
√	Generalization of the conclusions	Discussed in the context of the results.
√	Guidelines for future research	We recommend nesting analysis within arthroplasty registers as well as definitive randomised controlled trials
√	Disclosure of funding source	In "Acknowledgement" section

eTable 3. Literature search strategy

Relevant studies, published from inception to November 2018 (date last searched), were identified through electronic searches limited to the English language using MEDLINE, EMBASE, Web of Science, and Cochrane databases. Electronic searches were supplemented by scanning reference lists of articles identified for all relevant studies (including review articles) and by hand searching of relevant journals.

Database: Ovid MEDLINE(R) <1946 to present>
Search Strategy:
----
1 exp Knee Prosthesis/ (10710)
2 exp Arthroplasty, Replacement, Knee/ (20199)
3 exp Knee Joint/ (55210)
4 fixation.mp. (197432)
5 cement*.mp. (65551)
6 uncemented.mp. (2661)
7 hybrid.mp. (149302)
8 reverse hybrid.mp. (33)
9 stem.mp. (416609)
10 exp Prosthesis-Related Infections/ (10888)
11 prosthetic joint infection.mp. (1011)
12 prosthetic infection.mp. (399)
13 exp Wound Infection/ (44055)
14 deep infection.mp. (2795)
15 exp SEPSIS/ (113415)
16 surgical site infection*.mp. (8323)
17 1 or 2 or 3 (72671)
18 4 or 5 or 6 or 7 or 8 or 9 (811858)
19 10 or 11 or 12 or 13 or 14 or 15 or 16 (170789)
20 17 and 18 and 19 (534)
21 limit 20 to humans (529)

****

Each part was specifically translated for searching the other databases (EMBASE, Web of Science, and Cochrane databases)

Supplementary Table S4. Reference list of included studies

1. Wilson MG, Kelley K, Thornhill TS. 1990. Infection as a complication of total knee-replacement arthroplasty. Risk factors and treatment in sixty-seven cases. J Bone Joint Surg Am 72:878-883.
2. Duffy GP, Berry DJ, Rand JA. 1998. Cement versus cementless fixation in total knee arthroplasty. Clin Orthop:66-72.
3. McCaskie AW, Deehan DJ, Green TP, et al. 1998. Randomised, prospective study comparing cemented and cementless total knee replacement: results of press-fit condylar total knee replacement at five years. J Bone Joint Surg Br 80:971-975.
4. Pecina M, Djapic T, Haspl M. 2000. Survival of cementless and cemented porous-coated anatomic knee replacements: retrospective cohort study. Croat Med J 41:168-172.
5. Eveillard M, Mertl P, Tramier B, et al. 2003. Effectiveness of gentamicin-impregnated cement in the prevention of deep wound infection after primary total knee arthroplasty. Infect Control Hosp Epidemiol 24:778-780.
6. Baker PN, Khaw FM, Kirk LM, et al. 2007. A randomised controlled trial of cemented versus cementless press-fit condylar total knee replacement: 15-year survival analysis. J Bone Joint Surg Br 89:1608-1614.
7. Beaupre LA, al-Yamani M, Huckell JR, et al. 2007. Hydroxyapatite-coated tibial implants compared with cemented tibial fixation in primary total knee arthroplasty. A randomized trial of outcomes at five years. J Bone Joint Surg Am 89:2204-2211.
8. Jamsen E, Huhtala H, Puolakka T, et al. 2009. Risk factors for infection after knee arthroplasty. A register-based analysis of 43,149 cases. J Bone Joint Surg Am 91-A:38-47.
9. Dowsey MM, Choong PF. 2009. Obese diabetic patients are at substantial risk for deep infection after primary TKA. Clin Orthop 467:1577-1581.
10. Gandhi R, Razak F, Pathy R, et al. 2009. Antibiotic bone cement and the incidence of deep infection after total knee arthroplasty. J Arthroplasty 24:1015-1018.
11. Namba RS, Chen Y, Paxton EW, et al. 2009. Outcomes of routine use of antibiotic-loaded cement in primary total knee arthroplasty. J Arthroplasty 24:44-47.
12. Demey G, Servien E, Lustig S, et al. 2011. Cemented versus uncemented femoral components in total knee arthroplasty. Knee Surg Sports Traumatol Arthrosc 19:1053-1059.
13. Namba RS, Inacio MC, Paxton EW. 2013. Risk factors associated with deep surgical site infections after primary total knee arthroplasty: an analysis of 56,216 knees. Journal of Bone & Joint Surgery - American Volume 95:775-782.
14. Lass R, Kubista B, Holinka J, et al. 2013. Comparison of cementless and hybrid cemented total knee arthroplasty. Orthopedics 36:e420-427.
15. Pelt CE, Gililland JM, Doble J, et al. 2013. Hybrid total knee arthroplasty revisited: midterm followup of hybrid versus cemented fixation in total knee arthroplasty. Biomed Res Int 2013:854871.
16. Hinarejos P, Guirro P, Leal J, et al. 2013. The use of erythromycin and colistin-loaded cement in total knee arthroplasty does not reduce the incidence of infection: a prospective randomized study in 3000 knees. Journal of Bone & Joint Surgery - American Volume 95:769-774.
17. Qadir R, Sidhu S, Ochsner JL, et al. 2014. Risk stratified usage of antibiotic-loaded bone cement for primary total knee arthroplasty: short term infection outcomes with a standardized cement protocol. J Arthroplasty 29:1622-1624.
18. Gutowski CJ, Zmistowski BM, Clyde CT, et al. 2014. The economics of using prophylactic antibiotic-loaded bone cement in total knee replacement. Bone Joint J 96-B:65-69.
19. Bohm E, Zhu N, Gu J, et al. 2014. Does adding antibiotics to cement reduce the need for early revision in total knee arthroplasty? Clinical Orthopaedics & Related Research 472:162-168.
20. Choy WS, Yang DS, Lee KW, et al. 2014. Cemented versus cementless fixation of a tibial component in LCS mobile-bearing total knee arthroplasty performed by a single surgeon. J Arthroplasty 29:2397-2401.
21. Lizaur-Utrilla A, Miralles-Munoz FA, Lopez-Prats FA. 2014. Similar survival between screw cementless and cemented tibial components in young patients with osteoarthritis. Knee Surg Sports Traumatol Arthrosc 22:1585-1590.
22. Petursson G, Fenstad AM, Havelin LI, et al. 2015. Better survival of hybrid total knee arthroplasty compared to cemented arthroplasty. Acta Orthop 86:714-720.
23. Wang H, Qiu GX, Lin J, et al. 2015. Antibiotic Bone Cement Cannot Reduce Deep Infection After Primary Total Knee Arthroplasty. Orthopedics 38:e462-466.
24. Fricka KB, Sritulanondha S, McAsey CJ. 2015. To Cement or Not? Two-Year Results of a Prospective, Randomized Study Comparing Cemented Vs. Cementless Total Knee Arthroplasty (TKA). J Arthroplasty 30:55-58.
25. Tayton ER, Frampton C, Hooper GJ, et al. 2016. The impact of patient and surgical factors on the rate of infection after primary total knee arthroplasty: an analysis of 64,566 joints from the New Zealand Joint Registry. Bone Joint J 98-B:334-340.
26. Wu CT, Chen IL, Wang JW, et al. 2016. Surgical Site Infection After Total Knee Arthroplasty: Risk Factors in Patients With Timely Administration of Systemic Prophylactic Antibiotics. J Arthroplasty 31:1568-1573.
27. Prudhon JL, Verdier R. 2017. Cemented or cementless total knee arthroplasty? - Comparative results of 200 cases at a minimum follow-up of 11 years. SICOT J 3:70.
28. Sanz-Ruiz P, Matas-Diez JA, Sanchez-Somolinos M, et al. 2017. Is the Commercial Antibiotic-Loaded Bone Cement Useful in Prophylaxis and Cost Saving After Knee and Hip Joint Arthroplasty? The Transatlantic Paradox. J Arthroplasty 32:1095-1099.
29. Vertullo CJ, Graves SE, Peng Y, et al. 2018. The effect of surgeon's preference for hybrid or cemented fixation on the long-term survivorship of total knee replacement. Acta Orthop 89:329-335.
30. Gwam CU, George NE, Etcheson JI, et al. 2018. Cementless versus Cemented Fixation in Total Knee Arthroplasty: Usage, Costs, and Complications during the Inpatient Period. J Knee Surg.

31. Miller AJ, Stimac JD, Smith LS, et al. 2018. Results of Cemented vs Cementless Primary Total Knee Arthroplasty Using the Same Implant Design. J Arthroplasty 33:1089-1093.
32. Lenguerrand E, Whitehouse MR, Beswick AD, et al. 2018 (In Press). Risk factors associated with revision for prosthetic joint infection (PJI) following knee replacement: an observational cohort study from the National Joint Registry for England, Wales, Northern Ireland and the Isle of Man. Lancet Infect Dis.

Supplementary Figure S1. Assessment of risk of bias in randomised controlled trials

	Randon sequence generation	*Allocation concealment*	*Blinding of participants & personnel*	*Blinding of outcome assessments*	*Incomplete outcome data*	*Selective reporting*	*Other bias*
McCaskie, 1998	-	-	-	-	+	+	-
Beaupre, 2007	+	+	-	+	+	+	-
Baker, 2007	-	-	-	-	+	+	-
Demey, 2011	+	+	+	-	+	+	-
Hinarejos, 2013	+	?	-	-	+	+	-
Choy, 2014	+	?	+	+	+	+	-
Lizaur-Utrilla, 2014	+	?	+	+	+	+	-
Fricka, 2015	+	?	-	-	+	+	-

+	Low risk of bias
?	Unclear risk of bias
-	High risk of bias

Supplementary Figure S2. Comparison of all cemented fixation with uncemented fixation and the risk of prosthetic joint infection in observational studies

Author, year of publication	No. of patients/knees	No. of PJIs	RR (95% CI)
Jamsen, 2009	40,135	387	1.51 (0.82, 2.79)
Prudhon, 2017	200	1	0.33 (0.01, 8.09)
Duffy, 1998	106	1	0.31 (0.01, 7.43)
Gwam, 2018	167,930	NR	2.31 (0.07, 71.24)
Pecina, 2000	142	4	1.52 (0.16, 14.17)
Lenguerrand, 2018	679,010	3,659	0.71 (0.60, 0.84)
Miller, 2018	400	1	3.00 (0.12, 73.21)
Wilson, 1990	4,171	65	1.78 (0.25, 12.51)
Overall			0.76 (0.64, 0.89)

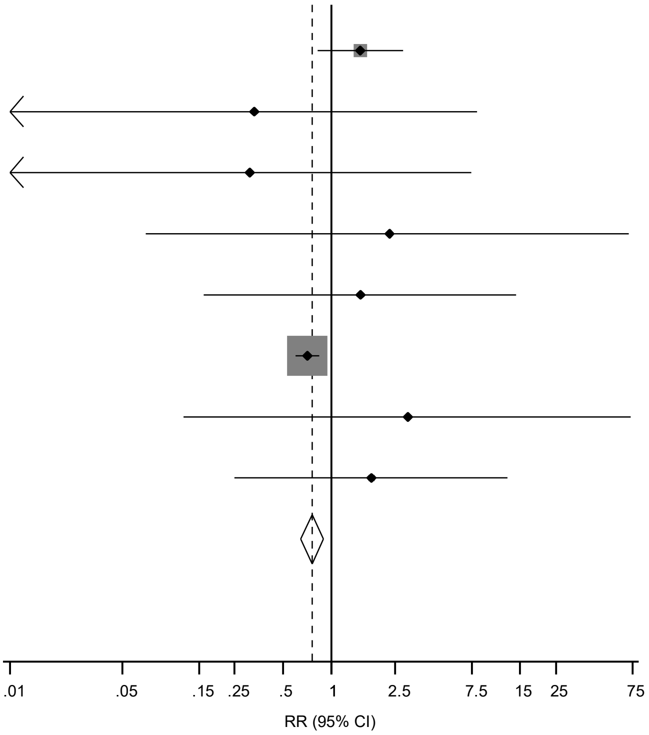

CI, confidence interval (bars); PJI, prosthetic joint infection; RR, relative risk

Supplementary Figure S3. Comparison of hybrid fixation with uncemented or all cemented fixations and the risk of prosthetic joint infection in observational studies

Author, year of publication	No. of patients/knees	No. of PJIs	RR (95% CI)
**Hybrid vs Cemented**			
Pertursson, 2015	24,680	217	0.81 (0.56, 1.17)
Vertullo, 2018	39,623	215	1.00 (0.73, 1.38)
Jamsen, 2009	40,135	387	1.26 (0.79, 2.01)
Pelt, 2013	304	5	0.86 (0.15, 5.08)
Pecina, 2000	142	2	4.00 (0.27, 59.04)
Wilson, 1990	4,171	66	0.91 (0.22, 3.66)
Subtotal			0.98 (0.80, 1.21)
**Uncemented vs Hybrid**			
Lass, 2013	120	1	0.33 (0.01, 8.02)
Wilson, 1990	4,171	3	1.97 (0.18, 21.12)
Subtotal			1.08 (0.16, 7.51)

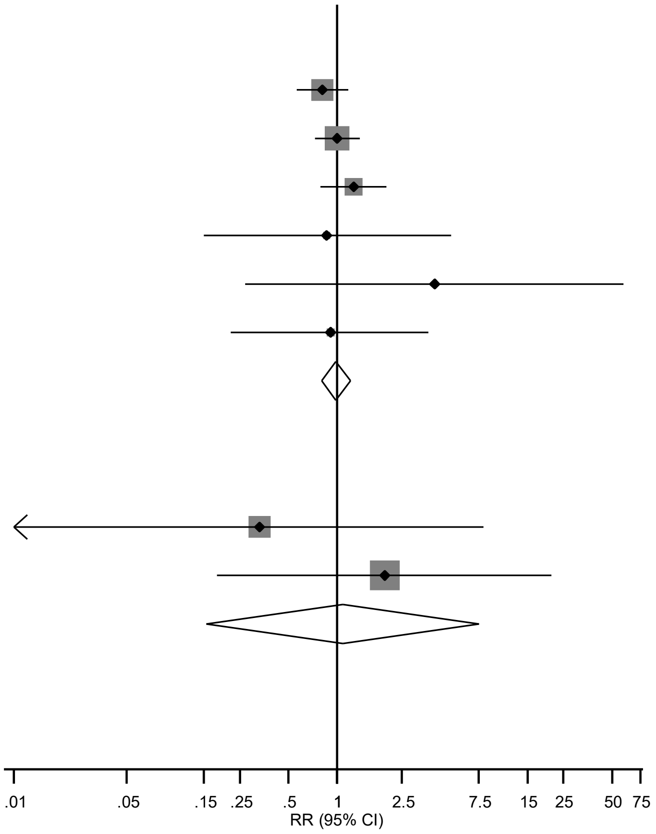

CI, confidence interval (bars); PJI, prosthetic joint infection; RR, relative risk

Supplementary Figure S4. Comparison of antibiotic-loaded cemented fixations with plain cemented fixations and the risk of prosthetic joint infection in observational studies

Author, year of publication	No. of patients/knees	No. of PJIs	RR (95% CI)
Wang, 2015	2,293	10	0.84 (0.11, 6.71)
Qadir, 2014	2,511	17	0.78 (0.30, 2.00)
Gutowski, 2014	7,878	63	1.10 (0.66, 1.83)
Bohm, 2014	36,681	36	1.89 (0.97, 3.69)
Namba, 2013	56,216	404	1.53 (1.18, 1.98)
Dowsey, 2009	1,214	18	1.05 (0.35, 3.11)
Gandhi, 2009	1,625	43	0.72 (0.39, 1.30)
Namba, 2009	22,889	182	1.70 (1.10, 2.50)
Eveillard, 2003	167	9	0.14 (0.02, 1.14)
Sanz-Ruiz, 2017	1,250	30	0.38 (0.16, 0.88)
Wu, 2016	3,152	48	0.33 (0.17, 0.64)
Tayton, 2016	64,566	179	1.18 (0.86, 1.62)
Overall			0.95 (0.69, 1.31)

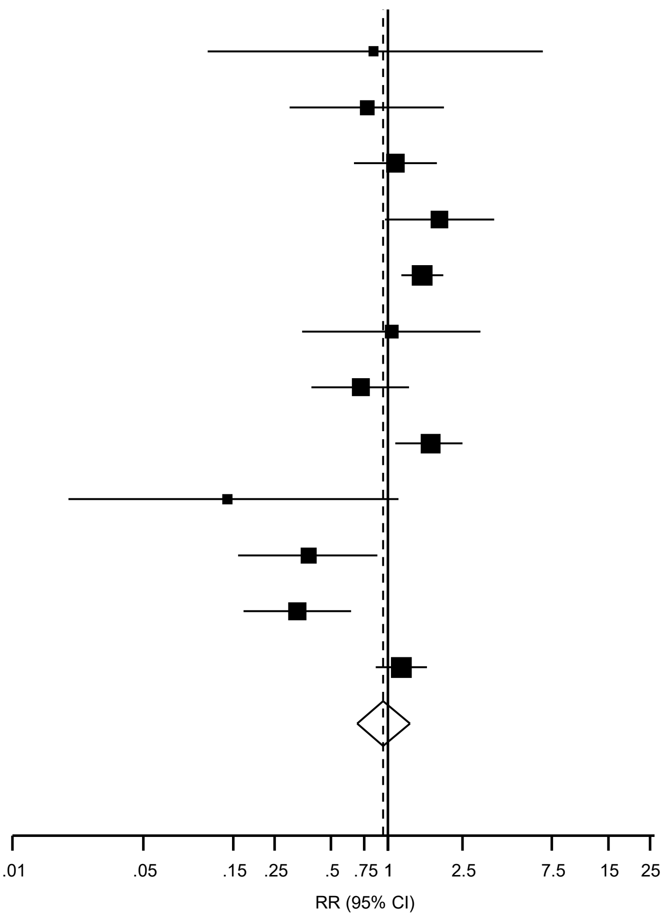

CI, confidence interval (bars); PJI, prosthetic joint infection; RR, relative risk

Supplementary Figure S5. Comparison of uncemented fixation with cemented or hybrid fixations and the risk of prosthetic joint infection in randomised controlled trials

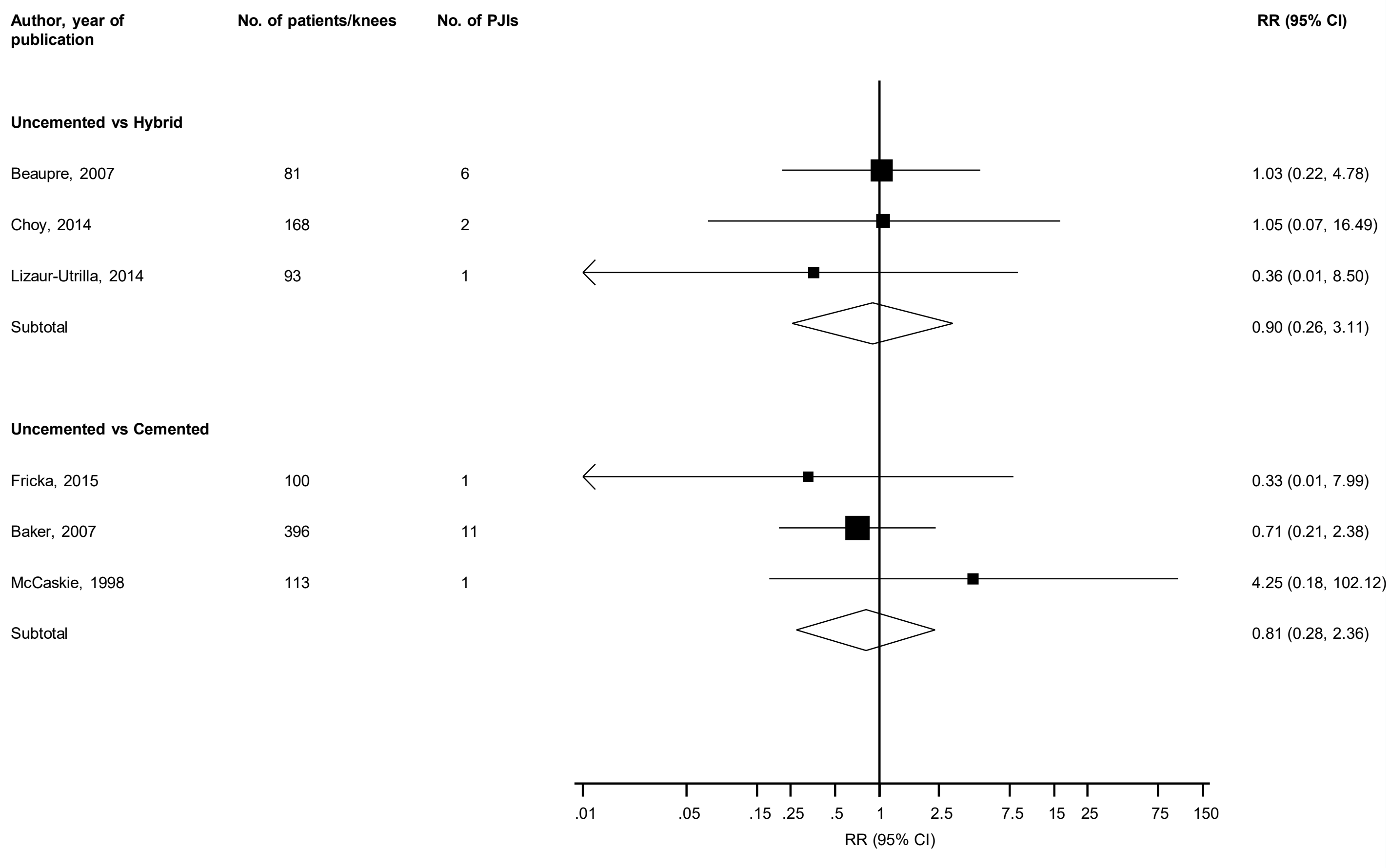

CI, confidence interval (bars); PJI, prosthetic joint infection; RR, relative risk

Supplementary Figure S6. Assessment of small study effects by funnel plots and Egger's regression symmetry tests

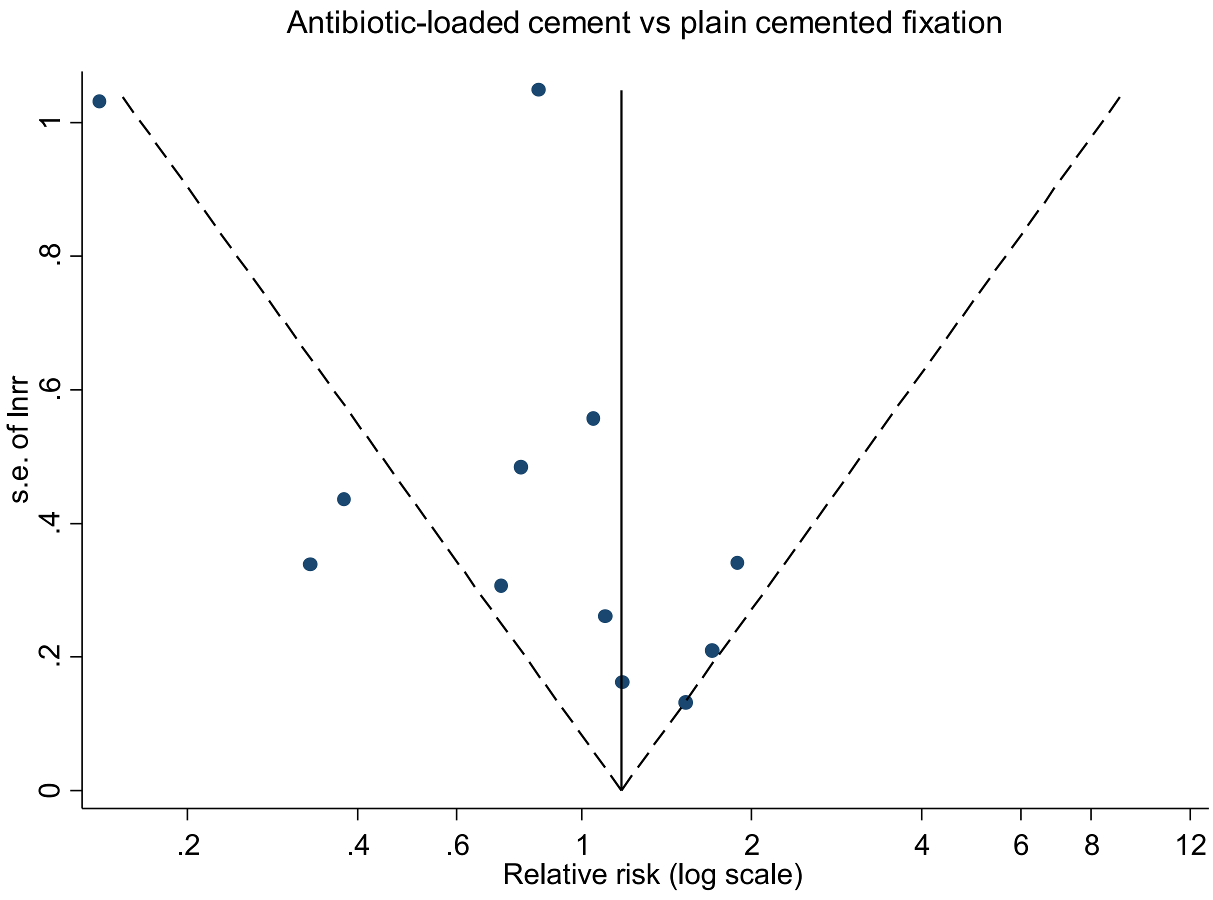

Supplement: Supplementary file 1 [file jcm-08-00828-s001.pdf]
